# On Ethically Solvent Leaders: The Roles of Pride and Moral Identity in Predicting Leader Ethical Behavior

**DOI:** 10.1007/s10551-016-3180-0

**Published:** 2016-05-03

**Authors:** Stacey Sanders, Barbara Wisse, Nico W. Van Yperen, Diana Rus

**Affiliations:** 1 Department of Work and Organizational Psychology, University of Groningen, Grote Kruisstraat 2/1, 9712 TS Groningen, The Netherlands; 2 Durham Business School, Durham University, Mill Hill Lane, Durham, DH1 3LB United Kingdom; 3 Creative Peas, IJburglaan 1026, 1087 JL Amsterdam, The Netherlands

**Keywords:** Authentic pride, Hubristic pride, Moral identity, Leader ethical behavior

## Abstract

The popular media has repeatedly pointed to pride as one of the key factors motivating leaders to behave unethically. However, given the devastating consequences that leader unethical behavior may have, a more scientific account of the role of pride is warranted. The present study differentiates between authentic and hubristic pride and assesses its impact on leader ethical behavior, while taking into consideration the extent to which leaders find it important to their self-concept to be a moral person. In two experiments we found that with higher levels of moral identity, authentically proud leaders are more likely to engage in ethical behavior than hubristically proud leaders, and that this effect is mediated by leaders’ motivation to act selflessly. A field survey among organizational leaders corroborated that moral identity may bring the positive effect of authentic pride and the negative effect of hubristic pride on leader ethical behavior to the forefront.

A recurring theme in corporate scandals, such as those at Enron and Olympus, is that leaders were focused on personal gain, while they lost sight of the needs and interests of others (van Gils et al. 2010). This resulted in leader behavior in which the balance of ethics tipped toward egoism at the expense of altruism (e.g., Bass and Steidlmeier 1999). Behavior that demonstrates social responsiveness to the needs and interests of others is generally considered to be ethical (e.g., Eisenberg 2000; Gilligan 1982; Kant 1785/1959),^1^ whereas a focus on egocentric needs and a lack of sensitivity to other people’s needs is considered to be unethical (Howell and Avolio 1992). Not surprisingly, leaders’ *unethical* behavior is associated with *negative outcomes*, such as employee workplace deviance (Thau et al. 2009), whereas leaders’ *ethical* behavior is related to *positive outcomes*, such as increased employee job satisfaction and organizational commitment (Schminke et al. 2005). Given the broad impact that leaders may have on the collective, it is essential to understand the conditions that prompt leaders to behave ethically.

Although scientific interest in ethical leader behavior has greatly intensified (e.g., Brown and Treviño 2006; Tenbrunsel and Smith-Crowe 2008; van Knippenberg et al. 2007), ethical behavior has primarily been viewed from a cognitive perspective (Haidt 2003; Reynolds 2006). Unfortunately, this cognitive perspective has been accompanied by a lack of attention to emotive determinants of ethical behavior (Reynolds and Ceranic 2007). Yet, advances in the study of ethical decision-making have highlighted the important role of moral emotions to ethical decision-making and behavior (e.g., Haidt 2001; Hoffman 2000; Pizarro 2000; Salvador and Folger 2009). The present study aims to contribute to the extant literature by looking at the largely overlooked role of moral emotions in predicting ethical leader behavior. In the context of ethical leadership, the emotion *pride* is of particular interest because individuals in leadership positions often feel proud (e.g., Bodolica and Spraggon 2010), and because pride may be expected to affect ethical behavior. We assert that authentic pride, the more pro-social facet of pride (Tracy and Robins 2007), will *motivate* leaders to act ethically, whereas hubristic pride, being the more anti-social facet (Wubben et al. 2012), will not.

Notably, recent research suggests that factors motivating leaders to act ethically will only translate into ethical behavior when being a moral person is important to their sense of self (i.e., when there is a direct implication to their identity; cf. Aquino et al. 2009; Brebels et al. 2010). We therefore contend that particularly with higher levels of leaders’ *moral identity*, authentically proud leaders will act more ethically than hubristically proud leaders. We further propose that leaders’ motivation to act ethically mediates this interactive effect of pride and moral identity on ethical leader behavior. As such, this research aims to contribute to our understanding of ethical leader behavior by providing an integrative account of emotions and moral identity that may explain why some leaders seem to function with a fully formed moral compass while others do not.

## Pride and Leader Ethical Behavior

Several scholars have highlighted the important role of discrete emotions to ethical decision-making and behavior (e.g., Haidt 2001; Pizarro 2000; Salvador and Folger 2009). Yet, not all emotions are considered equally relevant to ethical behavior, that is, some emotions are considered to have more “moral” connotations than others do. According to Haidt (2003), an emotion is a more “moral” emotion to the extent that it elicits pro-social action tendencies. Moral emotions can be powerful motivators, providing individuals with the desire to behave in an ethical manner (Kroll and Egan 2004). So far, research has largely focused on (negative) moral emotions, such as guilt and embarrassment (e.g., Eisenberg 2000; Keltner and Buswell 1997), and findings pertaining to their capability to elicit pro-social action tendencies go relatively undisputed. Yet, there is one emotion that sometimes elicits pro-social action tendencies and sometimes elicits anti-social tendencies, and that is the emotion of *pride*.

In fact, pride has been associated with both negative and positive *inter*personal consequences (Ashton-James and Tracy 2012; Leary 2007; Michie 2009; Spraggon and Bodolica 2015; Williams and DeSteno 2009). For instance, on the one hand, pride has been associated with leaders’ engagement in financial reporting frauds (Magnan et al. 2008). On the other hand, pride has also been depicted as an emotion motivating altruistic behavior on the part of leaders (Michie 2009). To solve pride’s paradoxical nature, researchers have made a theoretical as well as an empirical distinction between two facets of pride (Lewis 2000; Tracy and Robins 2007), with the one facet—*authentic* pride—being characterized by feelings of accomplishment and confidence, and the other facet—*hubristic* pride—being marked by arrogance and conceit.

These two facets of pride are differentially related to causal attributions,^2^ personality traits, and behavioral outcomes. *Authentic pride* is positively associated with skills enhancement, genuine self-esteem, perseverance at difficult tasks (Williams and DeSteno 2008), and pro-social personality traits, such as conscientiousness, agreeableness (Tracy and Robins 2007), and self-control (Carver et al. 2010). In contrast, authentic pride is negatively associated with anti-social personality traits, such as hostility and anger (Carver et al. 2010). Not only is authentic pride positively associated with pro-social personality traits, authentically proud people also behave more pro-socially. For instance, research shows that individuals who verbally express authentic pride, as compared to hubristic pride, are perceived as having acted more pro-socially (Wubben et al. 2012), and organizational leaders’ authentic pride has been positively related to their display of altruistic behavior (Michie 2009). In addition, it has been argued that authentic pride can serve as a self-regulatory mechanism that helps corporate leaders to govern their own social behavior (Spraggon and Bodolica 2015).

In contrast to authentic pride, *hubristic pride* is positively associated with self-enhancement, which can result in uncaring, exploitative behaviors toward others (Tracy et al. 2009). Moreover, hubristic pride is positively associated with anti-social personality traits, such as anger and aggression, whereas it is negatively associated with pro-social personality traits, such as agreeableness, conscientiousness, and self-control (Carver et al. 2010). Furthermore, previous research has positively associated hubristic pride with leader *un*ethical behavior such as leaders’ engagement in corporate illegal acts (Mishina et al. 2010).

Based on these findings, we argue that, of the two facets of pride, authentic pride is the true “moral emotion,” triggering a pro-social action tendency that provides the motivational ‘spark’ for leaders to act ethically. Specifically, we assert that authentically proud leaders are *motivated* to act selflessly and therefore also more likely to behave ethically, whereas hubristically proud leaders are less likely to take others’ welfare into account and to display ethical behavior.

## Pride, Moral Identity, and Leader Ethical Behavior

Interestingly, although emotional states generally do affect people’s behavior, the strength of this association has been found to be contingent on several intra-individual variables (e.g., Nelissen et al. 2007; Tanghe et al. 2010). A potential critical factor in this respect is the set of beliefs that people have about themselves, or their self-concept (cf. Hardy and Carlo 2005). A self-conception that is particularly relevant in the context of ethical behavior is one’s moral identity, defined as a self-conception organized around a set of moral traits (Aquino and Reed 2002).

The more central a person’s moral identity is to the sense of self, the more important it is to the person to be moral. Not surprisingly therefore, a growing body of research shows that moral identity is a powerful regulator and motivator of ethical (Detert et al. 2008; Hardy and Carlo 2005; Lapsley and Lasky 2001; Shao et al. 2008), and pro-social behavior (e.g., donating food to the needy, contribute to a public good; Aquino and Reed 2002). Moreover, research also shows that moral identity is positively associated with leaders’ display of ethical leadership (Mayer et al. 2012) and use of fair procedures (Brebels et al. 2010), and negatively impacts moral disengagement and the occurrence of unethical leader behavior (e.g., lying in business negotiations; Aquino and Reed 2002; Reed and Aquino 2003; Sage et al. 2006).

Important to the present discussion, moral identity may not only have a direct effect on ethical behavior. It has been suggested that moral identity is an important element in the transformation of a tendency or urge to act ethically into actual ethical behavior (cf. Hardy and Carlo 2005; Aquino et al. 2009). Accordingly, we argue that moral identity impacts the motivational and subsequent behavioral consequences of emotional experiences, in particular of those emotions that are self-conscious, like pride. In fact, it has been argued that a distinctive characteristic of self-conscious emotions is that they require the ability to focus attention on self-representations (i.e., to self-reflect; “I”), and that self-conscious emotions motivate behavioral action toward the goals embodied in these self-representations (Tracy and Robins 2007). Thus, particularly when we understand cognitively that ‘playing nice’ is the right thing to do, the psychological force of emotions like guilt and (authentic) pride will actually make us do so.

In addition, it has been argued that the capacity of (moral) emotions to contribute to moral motivation and (subsequent) moral behavior depends on the presence of moral concerns (cf. Blasi 1999). Phrased differently, emotions that motivate individuals to act ethically are more likely to translate into ethical behavior when it is essential for one’s self-identity to be a moral person. In contrast, when ethical behavior does not reflect on the self-concept, that is, when being a moral person is not important to the sense of self, moral drivers are less likely to translate into heightened motivation to act selflessly and into actual ethical behavior. As such, we hypothesize that:

### **Hypothesis 1a:**

With higher levels of moral identity, authentically proud leaders show higher levels of ethical behavior than hubristically proud leaders.

Although moral emotions and moral identity are often viewed as motivators of ethical behavior (Hardy 2006), and ethical intentions are often used as a proxy for ethical behavior (Kish-Gephart et al. 2010), relatively few studies have tested whether motivation to act ethically indeed mediates the relationship between moral motivators and ethical behavior. Drawing on Ajzen’s (1985, 1991) Theory of Planned Behavior, it can be argued that intentions—capturing the motivations for behavior—directly precede behavior. Hence, the stronger the person’ motivation for engaging in the behavior, the greater the likelihood that the actual behavior will be carried out. Meta-analytic results indeed show that intentions are significantly positively associated with actual behavior (Armitage 2001). In a similar vein, we argue that pride and moral identity interact to predict a leader’s motivation to act selflessly, and that this motivation in turn gives rise to actual ethical behavior. Specifically, we predict that:

### **Hypothesis 1b:**

The interactive effect of pride and moral identity on leader ethical behavior is mediated by the motivation to act selflessly.

## Overview of the Present Research

To investigate the combined effects of pride and moral identity on leader ethical behavior, we conducted two experimental studies (Study 1 and 2) and one field study (Study 3). We opted for a multiple-study, multiple-method approach in order to establish causality and to increase external validity. In both experimental studies, we induced feelings of pride (i.e., authentic vs. hubristic pride), measured the motivation to act selflessly, and assessed leader ethical behavior using *behavioral* measures. In Study 1, we measured participants’ self-importance of moral identity, and in Study 2, we manipulated the salience of participants’ moral identity. Both experimental studies are in particular suitable for the purposes of establishing causality between the manipulated factors and the outcome variables. Because we can only assume that the same relationships *could* exist outside the laboratory (Goodwin et al. 2000), in Study 3, we sought to bring the test of our hypotheses closer to a real-life setting by using a sample of organizational leaders. We measured leaders’ trait-like tendency to experience feelings of authentic and hubristic pride, leaders’ self-importance of moral identity, and their ethical behavior displayed in a work-context.

## Study 1

### Method

#### Participants and Design

Fifty-three undergraduate Dutch psychology students (15 males, 38 females) participated voluntarily in exchange for partial course credits or €8 (approximately US $12). Participants’ mean age was 20.04 years (SD = 2.22) and they were randomly assigned to one of the two pride conditions (authentic vs. hubristic pride). Moral identity centrality was added to the design as a continuous variable.

#### Procedure and Experimental Set-Up

Participants were invited to participate in a computer-mediated experiment and were seated in individual cubicles. They were told that the experiment consisted of two unrelated parts. In what was labeled “Study 1,” participants filled out some questionnaires including the self-importance of moral identity (henceforth *moral identity*) measure (Aquino and Reed 2002). Labeled as an independent “Study 2,” participants were informed that they were to work with another participant in a leader–follower relationship and that a network connection among participants would be established. In reality, interaction was simulated via the experimental set-up. Based on a purported leadership style test, *all* participants were assigned to the leader role. Moreover, *prior* to the task (which included an asymmetrical ultimatum game), participants completed the pride manipulation—allegedly to keep them busy while waiting for a connection to be established with their follower—and answered some questions. Finally, after answering some demographic indicators participants were debriefed, thanked, and paid for their participation.

#### Moral Identity Measure

*Moral identity* was measured using the five-item internalization subscale of Aquino and Reed’s (2002) validated self-importance of moral identity questionnaire. These items assess the extent to which moral trait associations are rooted in a person’s sense of self, and are previously shown to be internally consistent and to have a stable factor structure (Aquino and Reed 2002; Reed and Aquino 2003). Participants are presented with nine characteristics that describe a person (e.g., caring, compassionate, fair, and friendly), and are asked to visualize this person for a moment. Subsequently, participants respond to items including “Being someone who has these characteristics is an important part of who I am.” Responses on all five items were assessed using a 7-point Likert-type scale (1 = *strongly disagree*, 7 = *strongly agree*) and were averaged into a single moral identity score (*α* = .71, *M* = 5.86, SD = 0.59).

#### Task

Leader ethical behavior is often depicted as behavior that reflects the tension between egocentrism and responsiveness to the needs and interests of others (Bass and Steidlmeier 1999; Turner et al. 2002). Therefore, we adopted a paradigm in which both self-serving and other-serving behaviors are plausible options. Ultimatum games, characterized by the choice of acting in one’s self-interest or to sacrifice one’s interests to the benefit of others, represent such a paradigm (van Dijk and Vermunt 2000). Moreover, as business settings usually involve asymmetric information, with the allocator knowing more than the recipient (Ackert et al. 2011), we opted for an *asymmetrical ultimatum game* in the present study (e.g., Moran and Schweitzer 2008). The asymmetry of the game provides participants with the opportunity to act self-interested or selflessly outside the awareness of the follower. For instance, Moran and Schweitzer (2008) used this game to demonstrate that envy is associated with deception. In addition, the asymmetry of the game makes the division less likely to be influenced by impression management concerns.

As a leader, participants had to divide fifty lottery tickets between themselves and their follower. Participants were told that the follower would have the opportunity to either accept or reject the proposed division. If the proposed division would be accepted, both follower and leader would earn the amount proposed. If the follower would reject the offer, then both would earn nothing. Every ticket was counted as one lottery-entry for one of the three prizes (of 50, 20, and 10 Euros). Hence, the more tickets one obtained the higher the chances of winning one of these three prizes. Participants were told that the follower was not aware of the exact number of lottery tickets they had at their disposal. Allegedly, the follower thought that there were only twenty lottery tickets to divide instead of fifty, which gave participants the possibility to unobtrusively award more tickets to themselves. At the end of the experiment, three participants received a prize of 10, 20, or 50 Euros.

#### Pride Manipulation

Prior to the asymmetrical ultimatum game, pride was manipulated using a Relived Emotion Task in which thinking back of a time in which you experienced the emotion can induce the emotion in the present (Ekman et al. 1983; for a similar type of manipulation see Ashton-James and Tracy 2012). Specifically, participants were asked to vividly recall and to provide a written report of a particular incident in their lives where they experienced a feeling of authentic pride, or a feeling of hubristic pride.

In the *authentic pride condition* participants read:

Please recall a particular incident in which you felt really proud of your own behavior. That is, remember a situation in which you felt accomplished, fulfilled, and/or confident. In this situation you were very successful as a consequence of your own exertion, effort or hard work; a situation in which you excelled by trying hard.

In the *hubristic pride condition* participants read:

Please recall a particular incident in which you felt really proud of yourself. That is, remember a situation in which you felt stuck-up, conceited, and/or arrogant. In this situation you were very successful as a consequence of your own natural talent, intelligence or personality; a situation in which you excelled without even trying hard.

### Dependent Measures

#### Manipulation Checks

To assess the success of the pride manipulation, participants answered one multiple-choice question with three answer alternatives (i.e., “I was asked to describe a situation in which: I felt proud due to my own hard work and effort vs. my own natural talent, intelligence, or personality vs. none of these two alternatives”). Additionally, on a scale ranging from zero (*not at all*) to hundred (*completely*) participants indicated to what degree their feelings of pride could be attributed to their own effort (*M* = 80.63, SD = 12.41). A higher score is considered to indicate stronger authentic pride (cf. Carver et al. 2010; Tracy and Robins 2007).

#### Motivation to Act Selflessly

Participants’ motivation to act selflessly was measured with four items right after the asymmetrical ultimatum game. Specifically, participants were asked to think back about their motivation for the decision (i.e., “I was motivated to help the other person”; “I think the tickets should be distributed fairly”; “In the end, I was only focused on having as many lottery tickets as possible for myself” (*R*); “I wanted to make a strategic decision solely based on what is best for me” (*R*)) using a 7-point Likert-type scale (1 = *strongly disagree*, 7 = *strongly agree*, *α* = .86, *M* = 3.39, SD = 1.27).

#### Number of Tickets Awarded to Follower

The number of lottery tickets leaders awarded to their follower comprised our main dependent variable of leader ethical behavior (*M* = 14.15, SD = 6.15).

### Results

In all subsequently reported hierarchical regression analyses, we followed the guidelines of Aiken and West (1991). Pride was dummy coded (−.5 and .5 for hubristic pride and authentic pride, respectively) and moral identity was centered by subtracting the mean from each score. In Step 1, the main effects of the predictor variables (i.e., pride and moral identity) were entered into the analysis, in Step 2 the interaction effect was added.

#### Manipulation Checks

All participants answered the multiple-choice question correctly. Moreover, a hierarchical regression analysis on our source of pride score revealed, as expected, only a main effect of pride, *b* = 7.73, SE_*b*_ = 3.25, *t*(50) = 2.38, *p* = .02 (other *p*s > .13), with authentically proud individuals attributing their feelings of pride to a larger extent to their own effort (*M* = 84.50, SD = 12.49) than hubristically proud individuals (*M* = 76.60, SD = 11.19).

#### Number of Tickets Awarded to Follower

**Fig. 1:**
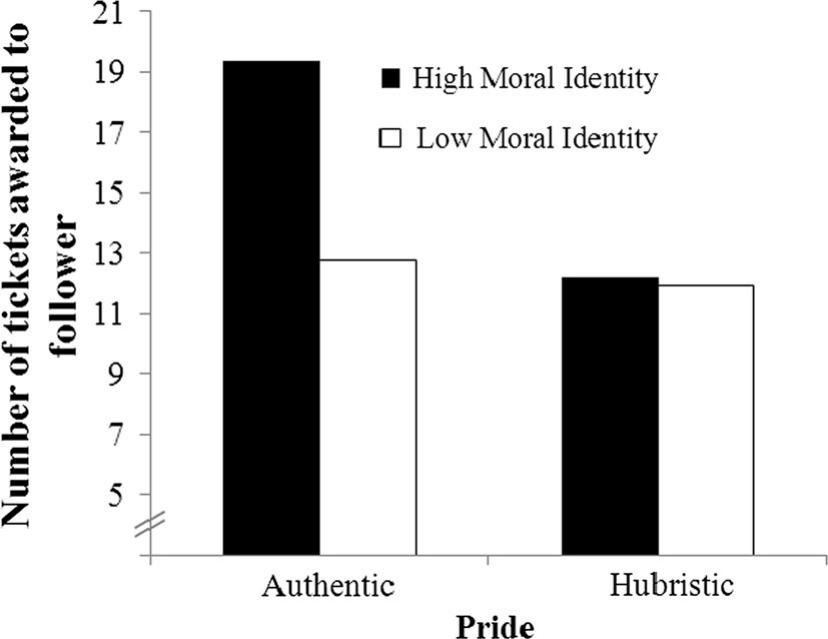
Number of tickets awarded to the follower as a function of pride and moral identity in Study 1

To test *Hypothesis 1a*, we conducted a hierarchical regression analysis on the number of lottery tickets leaders awarded to their follower. Step 1 explained a significant proportion of variance, Δ*R* ^2^ = .20, Δ*F* (2, 50) = 6.37, *p* = .03, and it unveiled a main effect of pride, *b* = 3.36, SE_*b*_ = 1.54, *t*(50) = 2.18, *p* = .03, indicating that authentically proud leaders (*M* = 15.89, SD = 7.56) acted more ethically than hubristically proud leaders (*M* = 12.35, SD = 3.57). We also found a main effect of moral identity, *b* = 3.60, SE_*b*_ = 1.32, *t*(50) = 2.18, *p* < .01, indicating that leaders for whom moral identity was central to their self-concept showed higher levels of ethical behavior. More importantly, Step 2 explained an additional significant proportion of variance in leader ethical behavior, ∆*R* ^2^ = .06, Δ*F* (1, 49) = 4.15, *p* = .05, and it revealed our predicted pride × moral identity interaction, *b* = 5.24, SE_*b*_ = 2.57, *t*(49) = 2.04, *p* = .05 (see Fig. 1). Follow-up analyses indicated that with higher levels of moral identity (1 SD above the mean), authentically proud leaders acted more ethically than hubristically proud leaders, *b* = 6.49, SE_*b*_ = 2.14, *t*(49) = 3.03, *p* < .01. Pride did not differentially impact ethical behavior for leaders with a low moral identity (1 SD below the mean), *b* = 0.30, SE_*b*_ = 2.12, *t*(49) = 0.14, *p* = .89.

#### Assessment of Conditional Indirect Effects

**Fig. 2:**
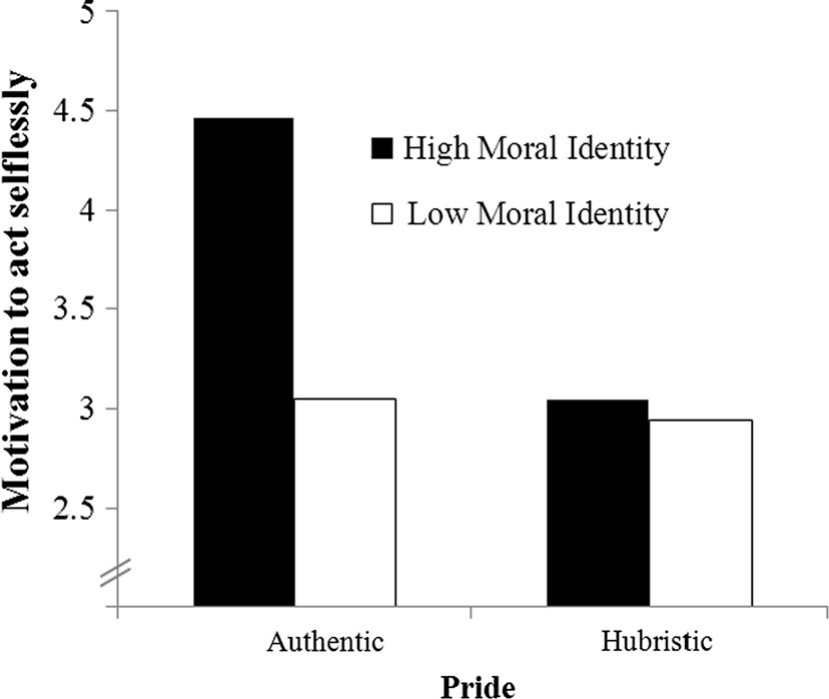
Motivation to act selflessly as a function of pride and moral identity in Study 1

Bootstrapped estimation of conditional indirect effects (see Preacher et al. 2007) was used to test *Hypothesis 1b*. Following the recommendations of Preacher et al. (2007), our analysis specified a mediated moderation model including three steps. In Step 1, we conducted a hierarchical regression analysis on the motivation to act selflessly to test whether the interaction of pride and moral identity influenced the mediator variable. This analysis revealed a significant main effect of pride, *b* = 0.75, SE_*b*_ = 0.32, *t*(50) = 2.36, *p* = .02, indicating that authentically proud leaders (*M* = 3.78, SD = 1.37) were more motivated to act selflessly than hubristically proud leaders (*M* = 2.99, SD = 1.03). We also found a main effect of moral identity, *b* = 0.68, SE_*b*_ = 0.27, *t*(50) = 2.50, *p* = .02, indicating that leaders with a high moral identity were more motivated to act selflessly. More importantly, we found a significant pride × moral identity interaction effect, *b* = 1.10, SE_*b*_ = 0.53, *t*(49) = 2.07, *p* = .04. Follow-up analyses revealed a pattern similar to our findings on leader ethical behavior (see Fig. 2). With higher levels of moral identity (1 SD above the mean), authentically proud leaders were more motivated to act selflessly than hubristically proud leaders, *b* = 1.41, SE_*b*_ = 0.44, *t*(49) = 3.18, *p* < .01. In contrast, with lower levels of moral identity (1 SD below the mean), pride did not differentially impact leaders’ motivation to act selflessly, *b* = 0.11, SE_*b*_ = 0.44, *t*(49) = 0.25, *p* = .80. This indicated that when moral identity was lower, authentically proud leaders acted as ethically as hubristically proud leaders (see also Fig. 1).

In Step 2, we found—in line with the strong positive correlation between the motivation to act selflessly and leader ethical behavior, *r* = .78, *p* < .001—that the motivation to act selflessly was positively associated with leader ethical behavior, *b* = 3.78, SE_*b*_ = 0.43, *t*(51) = 8.89, *p* < .001.

In Step 3, we tested the conditional indirect effects of pride via the motivation to act selflessly on leader ethical behavior for low levels of moral identity (1 SD below the mean) and high levels of moral identity (1 SD above the mean) separately. To assess these indirect effects, we used 5000 bootstrap samples and 95 % bias corrected and accelerated confidence intervals (BCa CI’s; Efron and Tibishirani 1993; Stine 1989). Bootstrapping confirmed that with higher levels of moral identity, authentically proud leaders act more ethically than hubristically proud leaders, through higher levels of motivation to act selflessly (*estimate*: 4.69; BCa CI: 1.69–8.95). Pride did not differentially impact leader ethical behavior via motivation to act selflessly for leaders with lower levels of moral identity (*estimate*: 0.19; BCa CI: −2.01 to 3.24).

## Study 2

The main goal of Study 2 was to replicate the findings of Study 1 with manipulations of both pride and moral identity. In addition, we aimed to extend the findings of Study 1 by adding a dependent measure to our design, namely the degree to which participants communicated honestly to their follower. Honesty is often considered to be the hallmark of ethical behavior (e.g., Aquino and Reed 2002; Lapsley and Lasky 2001), and, as such, constitutes another important indicator of leader ethical behavior.

### Method

#### Participants and Design

One hundred and fifteen undergraduate Dutch psychology students (23 males, 92 females) participated voluntarily in exchange for partial course credits. Participants’ mean age was 20.37 years (SD = 2.39) and they were randomly assigned to a 2 (pride: authentic vs. hubristic) × 2 (moral identity: salient vs. non-salient) between-subjects design.

#### Procedure and Experimental Set-Up

We followed the same procedure as in Study 1 with minor modifications. The main difference was the introduction of our moral identity manipulation. Moreover, we slightly adapted the asymmetrical ultimatum game (see van Dijk and Vermunt 2000, Experiment 1), to create the opportunity for participants to communicate (dis)honestly about their decision to the follower. Participants were asked to propose a division of 250 fiches to their follower, and they learned that the fiches were worth twice as much to them as to their follower (i.e., 0.2 lottery tickets per fiche vs. 0.1 lottery tickets per fiche). Importantly, participants were told that the follower was not aware of this differential value of the fiches. In addition to the number of fiches that were awarded to the follower (our dependent measure of leader ethical behavior in Study 1), in Study 2, we also asked the participants to write an e-mail to their follower in which they could elaborate on the proposed division (*Honesty of leaders’ communication to their follower*).

#### Moral Identity Manipulation

Prior to the asymmetrical ultimatum game, we manipulated moral identity using a computerized version of the manipulation developed by Aquino, Reed, Thau, and Freeman (2007). This manipulation has previously been shown to successfully activate moral identity within the working self-concept (Aquino et al. 2007, 2009; Reed et al. 2007). Participants were presented with a 9 × 5 matrix that contained nine words listed in the column of each row. In the salient moral identity condition, these words reflected moral traits (e.g., caring, compassionate); in the non-salient moral identity condition, these words denoted everyday household objects without moral content (e.g., book, chair). Participants were asked to type the words in the remaining four columns so that each participant typed in each of the words four times. Next, participants were instructed to take a few moments to think about each of these words, and to write a brief story about themselves with the use of these words.

#### Pride Manipulation

The pride manipulation was identical to the one used in Study 1.

### Dependent Measures

#### Manipulation Checks

To assess the success of our pride manipulation, we used the same measures as in Study 1. To check the success of our moral identity manipulation, we asked the participants to indicate the extent to which the following two statements accurately described their story (0 = *not at all*, 100 = *completely*): “In the story about myself, I depict myself as a moral person,” and “Moral characteristics are central in the story I wrote about myself” (*α* = .87, *M* = 52.39, SD = 27.82).

#### Motivation to Act Selflessly

The items were identical to the ones used in Study 1 (*α* = .72, *M* = 3.58, SD = 1.03).

#### Leader Ethical Behavior

Leader ethical behavior was indicated by the *number of fiches leaders awarded to their follower* (*M* = 123.71, SD = 15.33) and the *honesty of leaders’ communication to their follower* (*M* = 4.09, SD = 1.53). As part of the ultimatum game, participants were asked to inform their follower about the proposed division of fiches via the e-mail. Two independent raters, who were blind to the conditions, coded the content of all the e-mails for the degree of honesty. They used a 7-point scale that was accompanied by specific descriptions for each anchor (1 = *giving fal*se *information to the follower*, 7 = *telling the exact truth*). For example, “My fiches are worth twice as much as yours” was coded as seven, whereas “I will divide the fiches in half. Both you and I will then have an equal amount of fiches, and we will have the exact same chance of winning one of the three prizes” was coded as one. To make sure that both raters applied the same standards, both raters independently coded a subset of fifteen e-mails, and afterward discussed their ratings with each other. The independent coding of the remaining e-mails resulted in a high inter-rater agreement, Kendall’s *W* = .94, and the scores of the two independent raters were averaged into a single score.

### Results

In all analyses of variance (ANOVAs), pride (authentic vs. hubristic) and moral identity (salient vs. non-salient) were factors in the design.

#### Manipulation checks

First, 96.5 % of the participants answered the multiple-choice question regarding the pride manipulation correctly.^3^ Second, a two-way ANOVA revealed only a main effect of pride on our source of pride score, *F*(1, 111) = 258.60, *p* < .001, *η*_*p*_^2^ = .70 (other *p*s > .13), indicating that authentically proud individuals attributed their feelings of pride to a larger extent to their own effort (*M* = 79.63, SD = 19.59) than hubristically proud individuals (*M* = 25.57, SD = 16.73). A two-way ANOVA on our moral identity score, revealed only a main effect of moral identity, *F*(1, 111) = 92.54, *p* < .001, *η*_*p*_^2^ = .46 (other *p*s > .29), with participants in the salient moral identity condition scoring higher (*M* = 70.94, SD = 18.63) than participants in the non-salient moral identity condition (*M* = 33.53, SD = 22.44). We conclude that our manipulations were successful.

#### Number of Fiches Awarded to Follower

A two-way ANOVA on the number of fiches leaders awarded to their follower revealed a main effect of pride, *F*(1, 111) = 5.35, *p* = .02, *η*_*p*_^2^ = .05, showing that authentically proud leaders acted more ethically (*M* = 127.16, SD = 16.43) than hubristically proud leaders (*M* = 120.55, SD = 13.62). In line with the results of Study 1, this main effect was qualified by our predicted pride × moral identity interaction, *F*(1, 111) = 4.21, *p* = .04, *η*_*p*_^2^ = .04. Simple main effects analysis indicated that in the salient moral identity condition authentically proud leaders acted more ethically (*M* = 131.52, SD = 19.78) than hubristically proud leaders (*M* = 119.45, SD = 10.00), *F*(1, 111) = 9.65, *p* < .01, *η*_*p*_^2^ = .08. In the non-salient moral identity condition, no differential effects for pride were found, *F*(1, 111) = 0.03, *p* = .85.

#### Honesty of Leaders’ Communication to Their Follower

A two-way ANOVA on our honesty score controlling for the number of words leaders used in their e-mail to their follower, showed a marginally significant pride × moral identity interaction, *F*(1, 110) = 3.90, *p* = .051, *η*_*p*_^2^ = .03 (*p*s main effects > .26). In the salient moral identity condition authentically proud leaders were more honest (*M* = 4.62, SD = 1.46) than hubristically proud leaders (*M* = 3.67, SD = 1.67), *F*(1, 110) = 4.76, *p* = .03, *η*_*p*_^2^ = .04. No differential effects for pride were found in the non-salient moral identity condition, *F*(1, 110) = 0.34, *p* = .56.

#### Mediated Moderation

First, we conducted a mediated moderation analysis on the *number of fiches awarded to the follower*. In Step 1, we tested whether the interaction of pride and moral identity influenced the motivation to act selflessly. A two-way ANOVA on the motivation to act selflessly revealed a significant pride × moral identity interaction, *F*(1, 111) = 4.85, *p* = .03, *η*_*p*_^2^ = .04. In Step 2, we found—in line with the positive correlation between the motivation to act selflessly and the number of fiches awarded to the follower, *r* = .50, *p* < .001—that the motivation to act selflessly was positively associated with the number of fiches awarded to the follower, *b* = 7.42, SE_*b*_ = 1.21, *t*(113) = 6.12, *p* < .001. In Step 3, bootstrapping confirmed that only in the salient moral identity condition (1 SD above the mean), authentically proud leaders awarded more fiches to their follower than hubristically proud leaders, through higher levels of motivation to act selflessly (*estimate*: 2.20; BCa CI: 0.33 to 4.83). Pride did not differentially impact leader ethical behavior via motivation to act selflessly in the non-salient moral identity condition (1 SD below the mean, *estimate*: −0.70; BCa CI: −2.63 to 0.82). This indicated that when moral identity was not salient, authentically proud leaders awarded their follower with approximately the same number of fiches as hubristically proud leaders (see also Fig. 2).

Second, we conducted a mediated moderation analysis on the *honesty of leaders’ communication to their follower*. The results of Step 1 are identical to the one described above. In Step 2, we found—in line with the positive correlation between the motivation to act selflessly and leaders’ honesty, *r* = .27, *p* < .01—that the motivation to act selflessly was positively associated with the honesty of leaders’ communication to their follower, *b* = 0.40, SE_*b*_ = 0.13, *t*(113) = 3.02, *p* = .003. In Step 3, bootstrapping confirmed that only in the salient moral identity condition authentically proud leaders were more honest than hubristically proud leaders, through higher levels of motivation to act selflessly (*estimate*: 0.11; BCa CI: 0.10–0.30). Pride did not differentially impact honesty via motivation to act selflessly in the non-salient moral identity condition (*estimate*: −0.03; BCa CI: −0.14 to 0.04).

#### Discussion Study 1 and 2

Studies 1 and 2 focused on the interactive effects of pride and moral identity in predicting leader ethical behavior. These two studies consistently provide first empirical evidence that with higher levels of moral identity, authentically proud leaders act more ethically (i.e., act in a more selfless and honest way) than hubristically proud leaders (*Hypothesis 1a*). Additionally, the results indicate that with higher moral identity, authentically proud leaders are more *motivated* to act selflessly than hubristically proud leaders, which, in turn, positively predicts ethical *behavior* (*Hypothesis 1b*).

Study 3 was designed to contribute to Study 1 and 2 in several ways. First, to increase external validity we gathered data on organizational leaders. Second, in Study 3, we measured leader ethical behavior with the ethical leadership scale (Brown et al. 2005), and, thereby, broadened the measure of leader ethical behaviors (e.g., fairness, trust, and other-serving behavior) as compared with the measures used in Study 1 and 2. Third, in Studies 1 and 2, we induced the emotional state-like experience of authentic or hubristic pride by having participants relive an incident in their lives in which they felt this way. However, Tracy and Robins (2007) showed that some people are more prone to experience feelings of authentic or hubristic pride than others. Hence, the experience of both forms of pride may have a trait-like as well as a state-like basis. Therefore, in Study 3, we measured leaders’ trait-like tendency to experience feelings of both authentic and hubristic pride.

Study 3 thus aims to assess the independent effects of leaders’ tendency to experience both authentic and hubristic pride on their ethical behavior as moderated by their moral identity. Based on the findings of Study 1 and 2, we anticipated that authentic pride would be positively associated with leader ethical behavior, but particularly so among high moral identifiers. Hubristic pride appears to diminish any pro-social tendencies that people may have (e.g., Tracy et al. 2009). As a consequence moral identity’s function as a transformer of lingering tendencies to act pro-socially into actual ethical behavior will only reveal itself when hubristic pride is low. Hence, with higher levels of moral identity, leader ethical behavior is more likely to the extent that feelings of hubristic pride are less strong. Specifically, we tested the following hypotheses in Study 3:

##### **Hypothesis 2a:**

With higher levels of moral identity, authentic pride is positively associated with leader ethical behavior.

##### **Hypothesis 2b:**

With higher levels of moral identity, hubristic pride is negatively associated with leader ethical behavior.

## Study 3

### Method

#### Procedure

The study was conducted online as a leadership survey among leaders with at least three direct subordinates. Respondents were recruited via Amazon.com’s Mechanical Turk. Previous research has shown that the data obtained via the online platform Mechanical Turk are at least as reliable as those obtained via traditional methods (e.g., Buhrmester et al. 2011).

#### Sample

One hundred and thirty-eight respondents (44 % women) completed the survey online in exchange for $1. Respondents’ ages ranged from 20 to 65 with an average of 32.93 years (SD = 9.04). Respondents’ average work experience was 13.51 years (SD = 8.53), average tenure in a supervisory position was 5.46 years (SD = 5.14), average tenure on the current job was 4.50 years (SD = 4.03), and average number of direct subordinates was 10.07 (SD = 10.41).

#### Measures

Unless stated otherwise, all responses were assessed using a 7-point Likert-type scale (1 = *strongly disagree*, 7 = *strongly agree*). *Authentic pride* was measured using Tracy and Robins (2007) seven-item trait authentic pride scale (e.g., “I generally feel accomplished”). *Hubristic pride* was measured using Tracy and Robins (2007) seven-item trait hubristic pride scale (e.g., “I generally feel snobbish”). Analogous to Study 1, leaders’ moral identity was measured with the five items of the moral identity internalization subscale (Aquino and Reed 2002).

The 10-item Ethical Leadership Scale (Brown et al. 2005) comprised our dependent measure of *leader ethical behavior*. The original items were slightly adapted for the purposes of the current study in which leaders were asked to rate themselves on their ethical leadership (e.g., “Discusses business ethics or values with employees” was changed to “I discussed business ethics or values with employees”). For each of the ten items respondents indicated the number of times they had performed the described behavior during the past year (1 = *never*, 2 = *rarely*, 3 = *sometimes*, 4 = *usually*, 5 = *always*). Means, standard deviations, reliabilities, and correlations for all study variables are displayed in Table 1.

**Table 1:** Means, standard deviations, reliabilities, and intercorrelations for Study 3

	*M*	SD	(1)	(2)	(3)	(4)
(1) Authentic pride	5.86	0.75	(.88)			
(2) Hubristic pride	1.90	0.84	.04	(.90)		
(3) Moral identity	6.37	0.74	.28**	−.24**	(.75)	
(4) Leader ethical behavior	4.09	0.48	.30***	−.38**	.51***	(.80)

*N* = 138. Cronbach’s alphas are displayed on the diagonal

* *p* < .05; ** *p* < .01; *** *p* < .001

### Results

Prior to conducting a hierarchical regression analysis, we performed a confirmatory factor analysis (CFA) on our predictor variable items (i.e., authentic pride, hubristic pride, and moral identity) as well as our dependent variable items (i.e., leader ethical behavior) using AMOS (Arbuckle and Wothke 1999). We defined and compared five different factor structures, ranging from a one-factor model in which all items were indicative of one larger factor, to a four-factor model in which each of the study variables was indicative of their own factor. The four-factor model seems to have better fit (CFI = .84, IFI = .84, RMSEA = .08, C.I. RMSEA .07–.09) [*χ* ^2^(371, *N* = 138) = 675.45, *p* < .001] than all other models,^4^ supporting the notion that our study variables were not only theoretically but also empirically distinct.

#### Leader Ethical Behavior

We conducted a hierarchical regression analysis to test *Hypothesis 2a* and *Hypothesis 2b*. Leader ethical behavior was predicted by main effect terms for our independent variables (authentic pride, hubristic pride, moral identity) at Step 1, the two-way interaction terms at Step 2, and the three-way interaction term at Step 3.

**Fig. 3:**
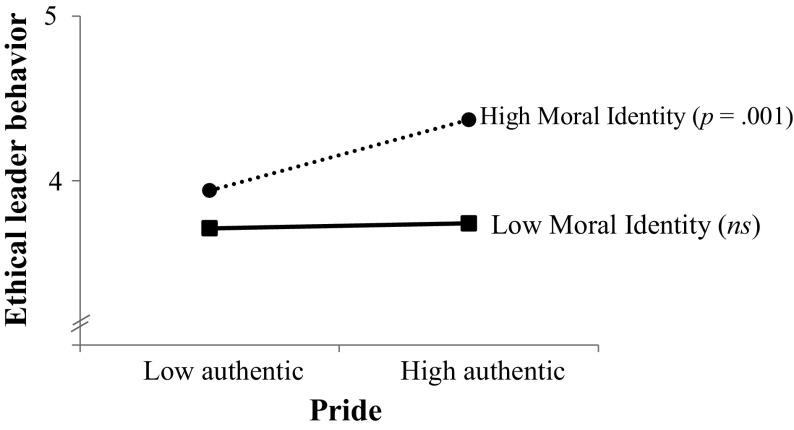
Leader ethical behavior as a function of leaders’ authentic pride and moral identity in Study 3

**Fig. 4:**
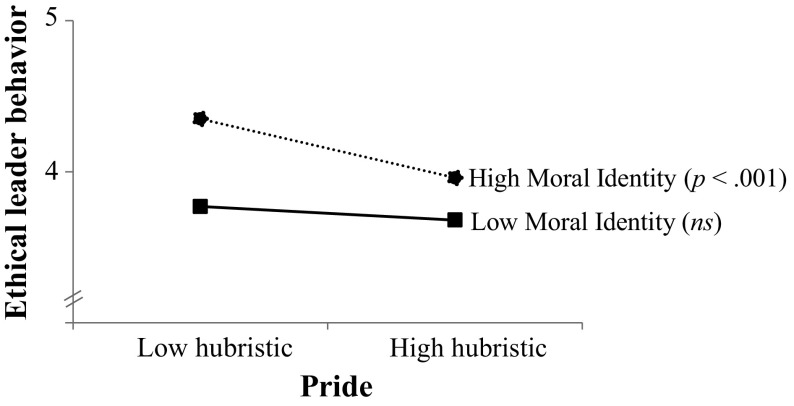
Leader ethical behavior as a function of leaders’ hubristic pride and moral identity in Study 3

Step 1 explained a significant proportion of variance and it unveiled the main effects of authentic pride, hubristic pride, and moral identity (see Table 2). Positively associated with leader ethical behavior were leaders’ trait-like authentic pride, *b* = .13, SE_*b*_ = 0.05, *t*(134) = 2.88, *p* < .01, and leaders’ moral identity, *b* = .25, SE_*b*_ = 0.05, *t*(134) = 5.13, *p* < .001. In contrast, leaders’ trait-like hubristic pride was negatively associated with ethical behavior, *b* = −.17, SE_*b*_ = 0.04, *t*(134) = −4.14, *p* < .001. More interestingly, Step 2 explained an additional significant proportion of variance in leader ethical behavior and it revealed our predicted authentic pride × moral identity, and hubristic pride × moral identity interactions (see also Table 2). In line with *Hypothesis* *2a*, authentic pride was positively associated with leader ethical behavior for leaders with a high moral identity (1 SD above the mean), *b* = .29, SE_*b*_ = 0.08, *t*(131) = 3.73, *p* < .001, but showed no relationship to ethical behavior for leaders with a low moral identity (1 SD below the mean), *b* = −.02, SE_*b*_ = 0.08, *t*(131) = −0.25, *p* = .80 (see Fig. 3). We also found empirical support for *Hypothesis* *2b*, hubristic pride was negatively associated with ethical behavior for leaders with a high moral identity, *b* = −.25, SE_*b*_ = 0.06, *t*(131) = −4.26, *p* < .001, but showed no relationship to ethical behavior for leaders with a low moral identity, *b* = −.08, SE_*b*_ = 0.06, *t*(131) = −1.22, *p* = .23 (see Fig. 4). These two-way interactions were not qualified by the three-way interaction in Step 4 (see Table 2).^5^

**Table 2:** Summary of regression analysis for authentic pride, hubristic pride, and moral identity (MI) predicting leader ethical behavior in Study 3

Variable	Step 1			Step 2			Step 3		
	*b*	SE_*b*_	*β*	*b*	SE_*b*_	*β*	*b*	SE_*b*_	*β*
Authentic pride	0.13	0.05	.21**	0.15	0.05	.24**	0.15	0.05	.23**
Hubristic pride	−0.17	0.04	−.30***	−0.16	0.04	−.28***	−0.14	0.04	−.24**
MI	0.24	0.05	.38***	0.29	0.05	.44***	0.29	0.05	.45***
Authentic × hubristic pride				0.06	0.06	.08	0.06	0.06	.07
Authentic pride × MI				0.18	0.07	.17*	0.17	0.07	.16*
Hubristic pride × MI				−0.12	0.06	−.14*	−0.12	0.06	−.14*
Authentic × hubristic × MI							−0.15	0.10	−.11
Δ*R* ^*2*^	.37			.04			.01		
*R* ^*2*^	.35			.38			.39		
*F*	26.05***			15.11***			13.41***		
*df*	134			131			120		

*N* = 138 (listwise)

* *p* < .05; ** *p* < .01; *** *p* < .001

### Discussion Study 3

Study 3 replicates the interactive effects observed in Study 1 and 2 by showing that with increasing importance of being a moral person to the self-concept, stronger feelings of authentic pride moves leaders toward higher levels of ethical behavior, whereas stronger feelings of hubristic pride moves leaders toward lower levels of ethical behavior. This replication with a different methodology, and a sample from a different country provides compelling evidence of the robustness of our findings.

## General Discussion

The rash of corporate scandals has instigated societal and scientific interest in (un)ethical leader behavior. Clearly, leaders’ lack of ethical conduct negatively impacts followers, organizations, and society at large. Hence, an increased understanding of the antecedents of leader ethical behavior, as well as an understanding of *when* and *why* these antecedents tap into leader ethical behavior is particularly crucial. In the present research, we aimed to contribute to the extant literature by taking an integrated approach by looking at the combined effects of pride and moral identity on leader ethical behavior.

We found first empirical evidence that particularly *when* moral identity is central to leaders’ sense of self (a) authentically proud leaders are more likely to engage in ethical behavior than hubristically proud leaders (Study 1 and 2; *Hypothesis 1a*), (b) authentic pride is positively related to leader ethical behavior (Study 3; *Hypothesis 2a*), and (c) hubristic pride is negatively related to leader ethical behavior (Study 3; *Hypothesis 2b*). Moreover, the finding that leaders’ motivation to act selflessly mediates the interactive effect of pride and moral identity on leader ethical behavior (Study 1 and 2; *Hypothesis 1b*) constitutes another unique contribution to the extant literature. Although we focused on *leader* ethical behavior in the current paper and leaders may be particularly apt to experience feelings of pride (Bodolica and Spraggon 2010), we suspect similar patterns for people who are not in a leadership position.

## Implications for the Study of Leader Ethical Behavior

The present study contributes to knowledge about the role of emotions and its interplay with moral identity in promoting leader ethical behavior in several ways. First, prior theorizing on the role of emotions in business ethics primarily focused on the role of general affect (Gaudine and Thorne 2001). Although a focus on general affective states in predicting ethical behavior can lead to important insights, our study shows that two different facets of the same emotion (i.e., authentic pride vs. hubristic pride) can already differentially impact leader ethical behavior. Specifically, we showed that of the two facets of pride, authentic pride is the more moral emotion. That is, in combination with higher levels of moral identity, authentic pride, not hubristic pride, promotes leader ethical behavior. As such, a systematic inquiry of how different *discrete* moral emotions impact leader ethical behavior may provide us with a more fine-grained picture of the influence of moral emotions on leader ethical behavior (cf. Angie et al. 2011 for a meta-analytic review on discrete emotions; Connelly et al. 2004; Treviño et al. 2006).

Second, historically, research has primarily focused on a cognitive approach to explaining ethical behavior (e.g., Reynolds 2006). Not surprisingly, therefore, scholars first started to investigate the influence of social cognitive factors, such as moral identity, on the link between cognitive antecedents and ethical behavior. For instance, Reynolds and Ceranic (2007) found that moral identity moderates the effects of moral judgment on moral behavior. However, moral emotions play at least an equally important role in explaining (un)ethical behavior as conscious reasoning (Haidt 2010). To our knowledge, no prior research has examined the moderating role of moral identity on the emotion-leader ethical behavior link. So, we are the first to demonstrate that moral identity is also critical in translating the pro-social action tendency triggered by feelings of authentic pride into actual ethical behavior. These findings illustrate that to fully understand the influence that emotions can have on leader ethical behavior an integrative account, combining research on moral emotions and more (social) cognitive factors, is necessary.

Third, our finding that leaders’ motivation to act selflessly can function as a mediator, is in line with both Ajzen’s (1985, 1991) Theory of Planned Behavior as well as the feeling-is-for-doing approach (Zeelenberg et al. 2008). This latter approach states that emotions motivate people in their decisions and subsequently guide their behaviors. However, motivation to act selflessly is not the only possible underlying mechanism that could link the interactive effects of pride and moral identity to leader ethical behavior. Although not addressed in the present study, moral emotions and moral identity alike are argued to increase moral awareness (e.g., DeCelles et al. 2012; Gino et al. 2011; Sumanth et al. 2011). Moral awareness refers to the identification of an issue as a moral one (Rest 1986), and, can be defined as “a person’s determination that a situation contains moral content and legitimately can be considered from a moral point of view” (Reynolds 2006, p. 233). As a result, moral awareness increases the likelihood that moral implications of one’s actions are taken into account, which could lead to subsequent adjustments in one’s behavior (DeCelles et al. 2012). Future research may focus on whether moral awareness indeed is another mediator variable linking the interactive effects of pride and moral identity to leader ethical behavior.

## Managerial Implications

On a more practical note, the present study provides some suggestions as to how to promote leader ethical behavior. We found that the tendency to experience feelings of authentic pride and to have a central moral identity positively relates to leader ethical behavior. To this end, organizations might benefit from including measures of leaders’ tendencies to experience feelings of authentic and hubristic pride, as well as a measure of leaders’ chronic self-importance of moral identity in their battery of leader selection criteria. As our findings denote, in terms of leader ethical behavior, organizations are likely to benefit from hiring leaders with high levels of authentic pride (or at least low levels of hubristic pride), and a highly central moral identity.

Moreover, as our manipulation of authentic and hubristic pride illustrates, emotions can be induced by recalling particular incidents in people’s lives, as well as by events, other people’s behavior and emotions, and social norms (see Lewis et al. 2008). The potential to induce emotions opens the door for cultivating or transforming the emotions experienced by leaders. Indeed, there is some research indicating that moral emotions can be educated (Maxwell 2008). Likewise, educating leaders on the influence of emotions—like pride—on ethical behavior might be a promising managerial tool for fostering leader ethical behavior. Moreover, in light of our results, combining interventions that are geared at fostering leaders’ authentic pride and curbing leaders’ hubristic pride with interventions focusing on strengthening leaders’ moral identity might prove to be particularly fruitful. One way to strengthen leaders’ moral identity is to provide them with the opportunities to act ethically. Acting ethically may bolster leaders’ moral identity, because it helps them to integrate morality in their self-identity (Damon 1984; Pratt et al. 2003).

## Strengths and Limitations

Inevitably, each of the study designs used to test our theoretical predictions has its own drawbacks. Therefore, a strength of the present research is the multiple-study, multiple-method approach in which the strengths of one method compensate for the limitations of the other method (Dipboye 1990). Studies 1 and 2 yielded experimental evidence with high internal validity, but could raise questions concerning external validity. In contrast, for Study 3, external validity poses less of a problem, but due to its correlational nature, it can be criticized for not providing evidence concerning causality.

The use of self-report measurements in Study 3 and the fact that all variables were assessed using a single questionnaire makes common method variance a potential problem. Although we acknowledge that the cross-sectional single-source design of Study 3 is suboptimal, previous research suggests that self-reports of undesirable behavior can be as accurate as more objective measures (Aquino and Douglas 2003; Hindelang et al. 1979). Moreover, the replication of our findings across studies employing different methodologies (i.e., two laboratory experiments and a field survey), measurements, and samples (i.e., Dutch students and business leaders in the United States) strengthens the confidence in our findings.

## Conclusion

The current findings highlight both the differential role of authentic pride and hubristic pride in predicting ethical behavior and the importance of integrating knowledge from research on (moral) emotions with research on social cognitive factors. By showing that authentic pride only motivates ethical behavior among high moral identifiers, we hope that the current findings inspire researchers to investigate the joint influence of emotive and (social) cognitive factors in explaining ethical behavior.

## Footnotes

1 In the current paper, we use the term ethical behavior to refer to behaviors in which the needs and interests of others are taken into consideration. As such, we adopt the term ethical behavior in our introduction and discussion section as an umbrella term for the several measures we used in the current paper: selfless behavior, honesty, and ethical leadership.

2 Note that there is some debate about the distinction between authentic and hubristic pride, especially when causal attributions are concerned. For a discussion see Holbrook et al. (2014) and Tracy and Robins (2014).

3 Analyses conducted on a sample excluding participants who did not answer the multiple-choice question correctly revealed largely identical results as the ones reported on the full sample.

4 The four-factor model has a better fit than the first three-factor model (CFI = .78, IFI = .78, RMSEA = .09, C.I. RMSEA .08–.10) [*χ* ^2^(375, *N* = 138) = 789.31, *p* < .001], ∆*χ* ^2^(4) = 113.86, *p* < .001, the second three-factor model (CFI = .54, IFI = .55, RMSEA = .13, C.I. RMSEA .12–.14) [*χ* ^2^(374, *N* = 138) = 1234.59, *p* < .001], ∆*χ* ^2^(3) = 559.14, *p* < .001, the two-factor model (CFI = .48, IFI = .49, RMSEA = .14, C.I. RMSEA .13–.15) [*χ* ^2^(377, *N* = 138) = 1349.00, *p* < .001], ∆*χ* ^2^(6) = 673.55, *p* < .001, or the one-factor model (CFI = .36, IFI = .37, RMSEA = .15, C.I. RMSEA .15–.16) [*χ* ^2^(378, *N* = 138) = 907.52, *p* < .001], ∆*χ* ^2^(7) = 232.07, *p* < .001.

5 Controlling for age, gender, and number of subordinates did not change the significance and direction of the results.
